# Laparoscopic appendectomy for metastatic cervical cancer presenting as appendicitis

**DOI:** 10.1186/s40792-021-01196-6

**Published:** 2021-05-07

**Authors:** Shota Fukai, Alan Kawarai Lefor, Ken Mizokami

**Affiliations:** 1 Department of Surgery, Tokyo Bay Medical Center, 3-4-32 Todaijima, Urayasu, Chiba 279-0001 Japan; 2 Department of Surgery, Jichi Medical University, 3311-1 Yakushiji, Shimotsuke-shi, Tochigi, 329-0498 Japan

**Keywords:** Appendiceal neoplasms, Metastasis, Cervical cancer

## Abstract

**Background:**

Metastatic lesions to the appendix are rare. They usually present with acute appendicitis or remain asymptomatic and are diagnosed incidentally. Metastases to the appendix have been reported from a number of primary tumor sites including ovary, colon, gastric and lung. We report a laparoscopic appendectomy for a metachronous metastatic lesion to the appendix from the uterine cervix.

**Case presentation:**

A 68-year-old woman, who underwent radical hysterectomy for cervical cancer 16 years previously, presented with nausea and gradually worsening right lower quadrant abdominal pain. Abdominal computed tomography scan showed an enlarged appendix and periappendiceal fat stranding. She was diagnosed with appendicitis and underwent laparoscopic appendectomy. Pathological findings showed adenocarcinoma in the submucosa and muscularis propria. Gastrointestinal endoscopy and positron emission tomography with computed tomography (PET–CT) did not show other lesions. Immunohistochemical analysis showed cytokeratin 7 (CK7) positive, cytokeratin 20 (CK20) negative, estrogen receptor (ER) 70–80% and progesterone receptor (PgR) 40–50%. The ER and PgR expression was similar to the cervical lesion 16 years previously, and the diagnosis was a metastatic lesion to the appendix from the uterine cervix.

**Conclusions:**

Metastasis to the appendix from cancer of the uterine cervix is a rare lesion.

## Background

Metastatic lesions to the appendix are extremely rare, and usually present with symptoms of acute appendicitis or remain asymptomatic and are diagnosed incidentally [1]. Previous reports of metastatic tumors to the appendix have included lesions from a number of primary sites including ovary, colon stomach and lung [2, 3], but metastases from cervical cancer were rarely reported. We report a laparoscopic appendectomy to treat a metastatic lesion to the appendix from primary cervical cancer.

## Case presentation

**Fig. 1:**
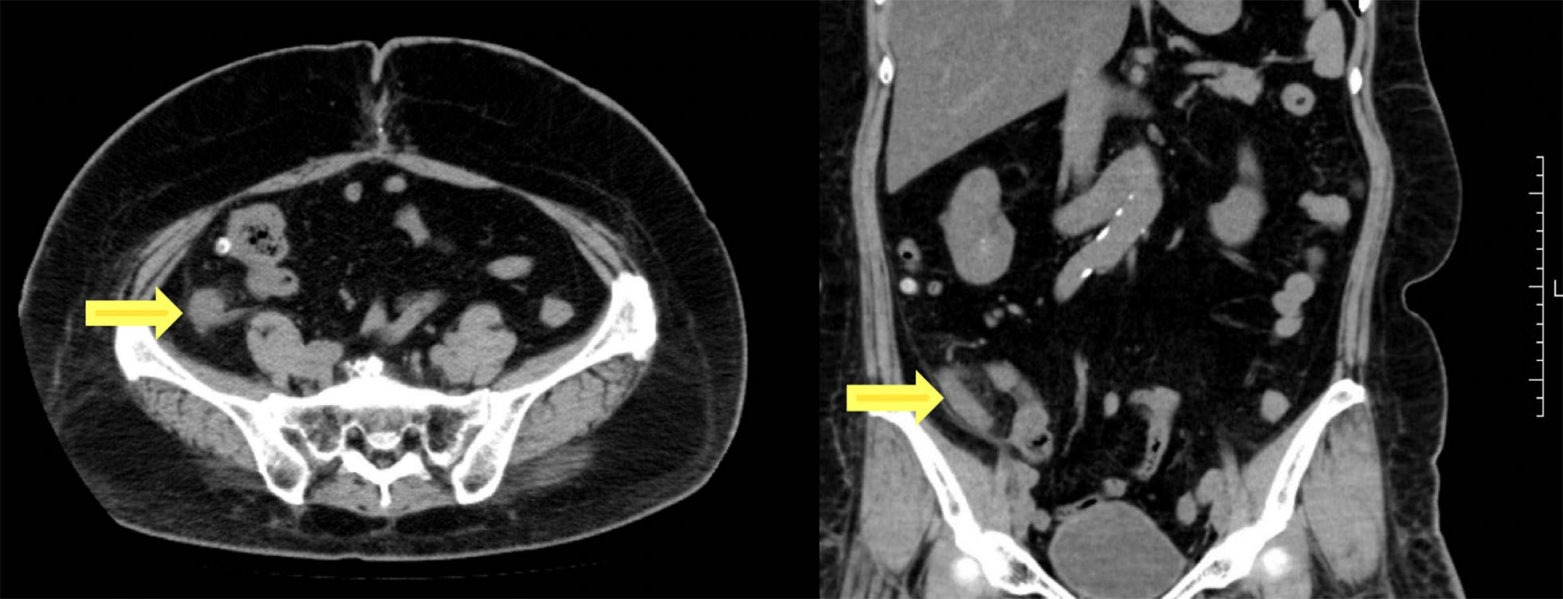
Preoperative computed tomography scan. Contrast-enhanced abdominal computed tomography scan in a 68-year-old woman who presented with nausea and gradually worsening right lower quadrant abdominal pain. The appendiceal diameter was enlarged 14 mm (yellow arrow) and periappendiceal fat stranding was observed

**Fig. 2:**
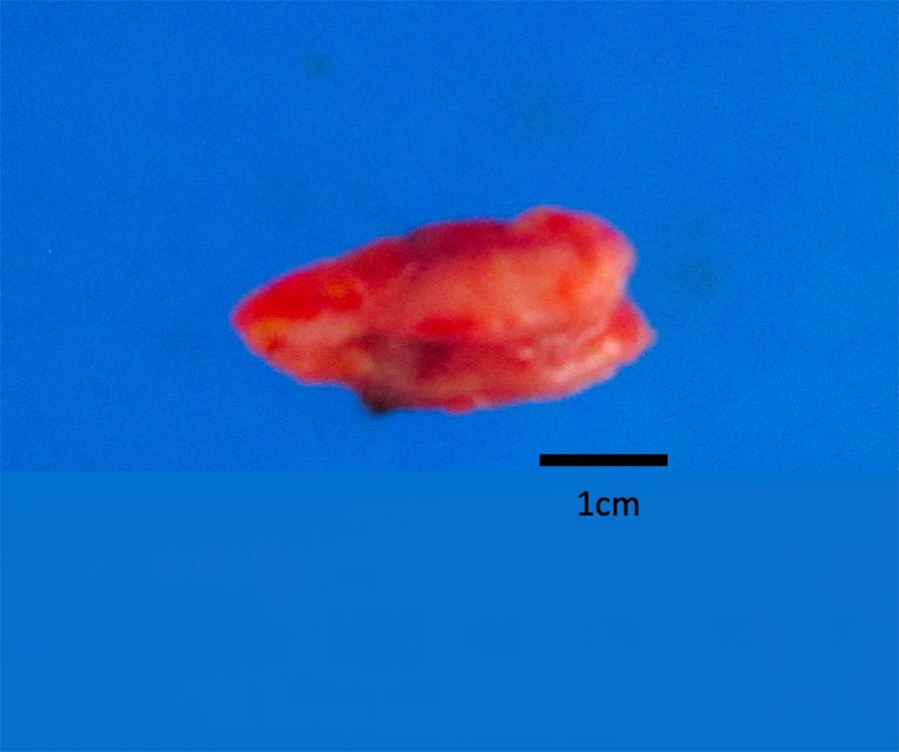
Gross findings of specimen. Macroscopic examination of the resected specimen showed that the wall was thickened and the mucosa was edematous and erythematous. There was no tumor palpable in the mucosa

**Fig. 3:**
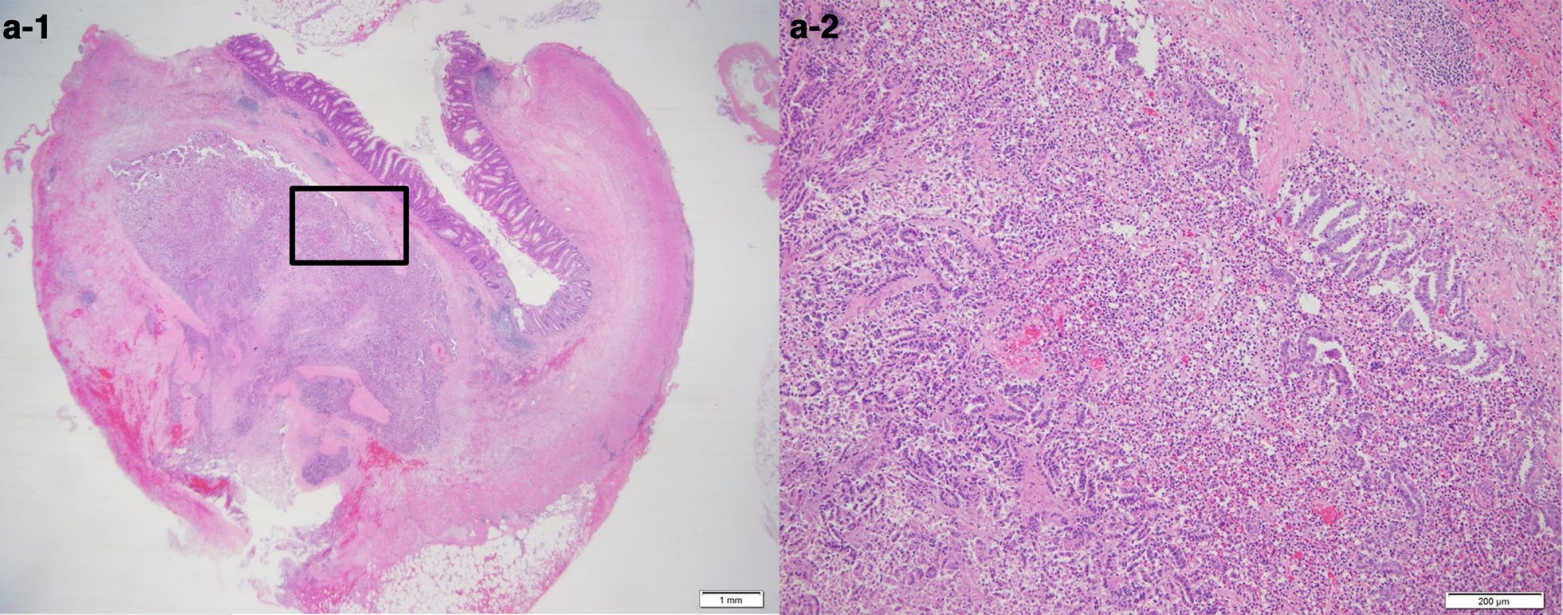
Pathological findings. Photomicrograph of the excised specimen at low magnification (hematoxylin and eosin stain) (**a-1**: × 1, **a-2**: × 10) showing adenocarcinoma in the submucosa and muscularis propria

**Fig. 4:**
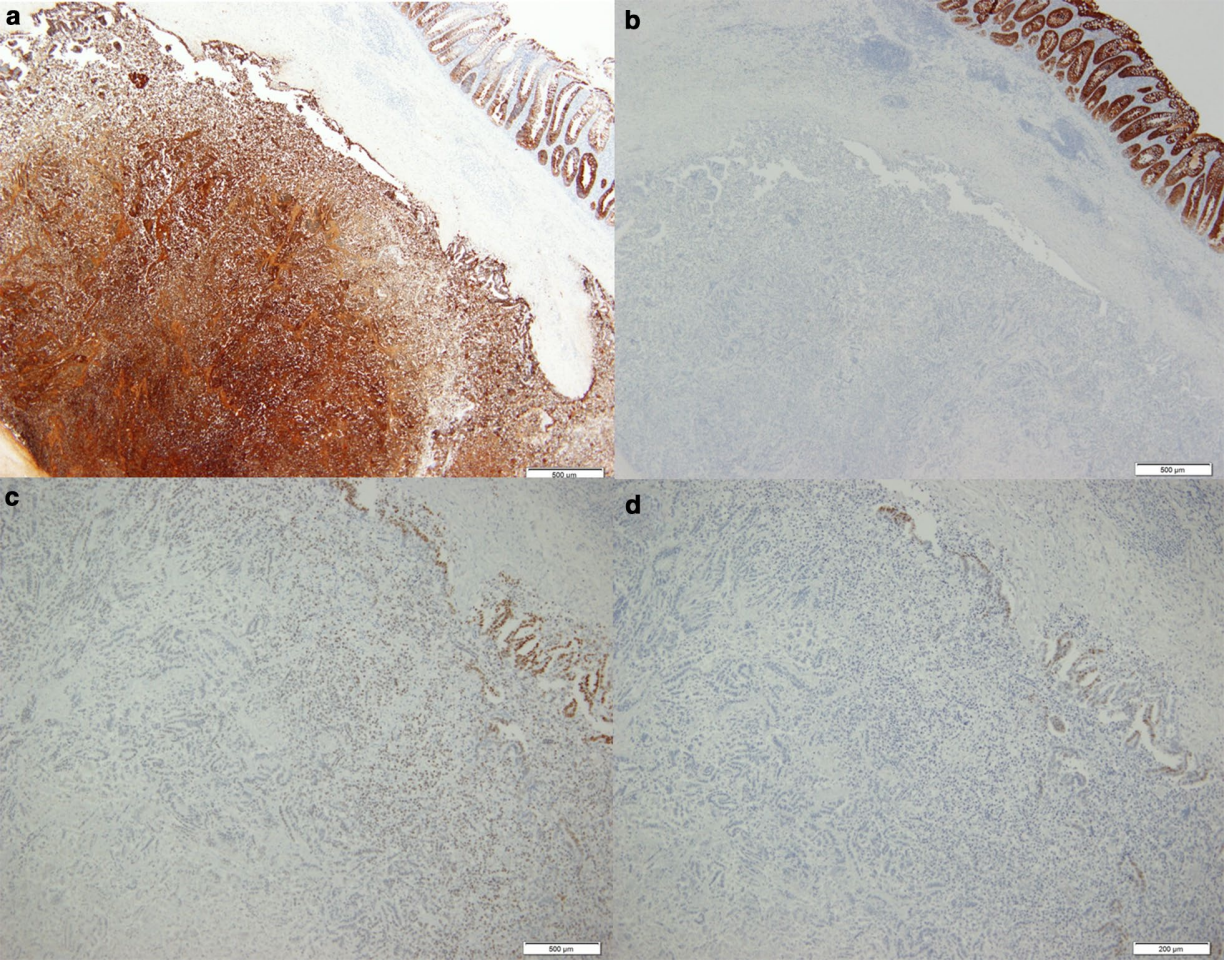
Immunohistochemical analysis. Immunohistochemical stains at low magnification (**a**: × 4, **b**: × 4, **c**: × 4, **d**: × 4) shows cytokeratin 7 (CK7) positive (**a**), cytokeratin 20 (CK20) negative (**b**), estrogen receptor (ER) 70–80% (**c**) and progesterone receptor (PgR) 40–50% (**d**)

**Fig. 5:**
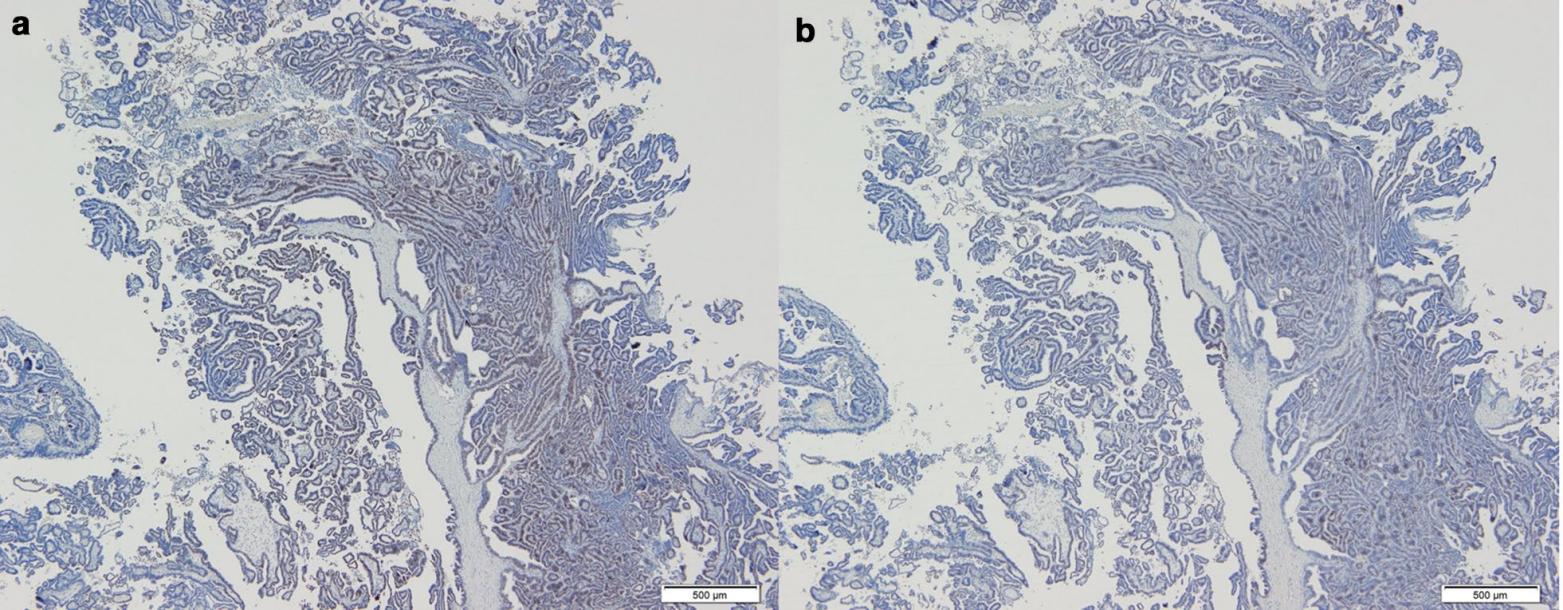
Immunohistochemical analysis of adenocarcinoma of the uterine cervix resected 16 years previously. Immunohistochemical stains (**a**: × 4, **b**: × 4) of the previous cervical lesion. The percentage of ER and PgR was 70–80% (**a**) and 40–50% (**b**), respectively, matching exactly the lesion in the appendix (Fig. 4)

A 68-year-old woman who underwent radical hysterectomy and adjuvant radiation therapy (total 50 Gy) for adenocarcinoma of the uterine cervix (pT1b, pN0, M0, pStage Ib (UICC8th)) 16 years previously, presented with nausea and gradually worsening right lower quadrant abdominal pain. Physical examination showed a blood pressure of 174/112 mmHg, heart rate of 76 beats/min, respiratory rate of 20 breaths/min, and normal temperature. Abdominal examination showed rebound tenderness in the right lower quadrant, consistent with localized peritonitis. Laboratory tests showed an elevated white blood cell count was 8600/μl. Abdominal computed tomography scan showed an enlarged appendix and periappendiceal fat stranding (Fig. 1). She was diagnosed with appendicitis and underwent laparoscopic appendectomy. The appendix was not grossly perforated and the tip adhered to the cecum. The appendix was resected in a retrograde manner and the specimen was 3 cm long. Macroscopic examination of the specimen showed that the wall was thickened and the mucosa was edematous and erythematous. There was no tumor palpable in the mucosa (Fig. 2). Histopathologic evaluation showed adenocarcinoma in the submucosa and muscularis propria suggesting a possible metastatic lesion (Fig. 3). Gastrointestinal endoscopy, gynecologic examination and positron emission tomography with computed tomography (PET–CT) scan found no evidence of malignancy. Immunohistochemical analysis showed cytokeratin 7 (CK7) positive, cytokeratin 20 (CK20) negative, estrogen receptor (ER) 70–80% and progesterone receptor (PgR) 40–50% (Fig. 4). The percentage of ER and PgR was similar to that in the cervical lesion resected 16 years previously (Fig. 5). The final diagnosis was adenocarcinoma of the uterine cervix metastatic to the appendix. The postoperative course was uneventful, and she was discharged on postoperative day 2.

## Discussion

Appendiceal tumors are very rare and metastatic lesions to the appendix are extremely uncommon. Metastases or direct invasion by a separate primary malignancy account for 77.2% of all pathologically confirmed appendiceal malignancies [4]. A metastatic lesion to the appendix may be diagnosed incidentally, but the increase in size of a metastatic lesion may cause stenosis or obstruction of the lumen of the appendix, which can lead to the development of appendicitis [5]. An extensive literature search identified just one report of squamous cell carcinoma of the uterine cervix with metastasis to the appendix. [6]. To the best of our knowledge, this is the first report of adenocarcinoma of the uterine cervix metastatic to the appendix.

Tumors of the appendix that are not obviously of appendiceal origin should prompt a search for other primary tumors. It is important to review the complete history, gastrointestinal endoscopy, gynecologic examination and PET–CT, because the most commonly reported lesions are from ovary, colon stomach and lung [2, 3]. In the case of a patient with a previous malignancy but a negative clinical examination and imaging studies, pathological findings from the previous malignancy may give important clues to the diagnosis. In the present patient, CK7 positive and CK20 negative immunohistochemical studies were consistent with her previous cervical cancer and the rate of ER and PgR positivity were the same as the previous cervical cancer, establishing the origin of the lesion found in the appendix.

The majority of cervical cancer recurrences are found within 2 years of the original diagnosis [7]. The most frequently observed metastatic sites are lung, para-aortic lymph nodes, abdominal cavity and supraclavicular lymph nodes [8]. In the present patient, metastasis to the appendix occurred 16 years after hysterectomy and irradiation, which is an extremely rare situation. To the best of our knowledge, there are no reports of a recurrence more than 10 years after treatment of cervical cancer. In fact, 89 to 99% of recurrences are detected within 5 years of treatment [9]. In the present patient, while the interval is unusually long, we believe that the histologic appearance and the immunohistochemistry strongly support the diagnosis of a metastatic lesion. Given the histologic and immunohistochemical evidence of a lesion found in the appendix that looks very different from appendiceal tissue and the same as her previous lesion, we believe that it is a metastatic lesion as described. It is conceivable that there could be another origin, although there is no evidence of such an origin. Kawabe et al. reported recurrent advanced gastric cancer diagnosed 20 years after partial gastrectomy and considered that delayed recurrence was related with the balance between cancer cell proliferation and apoptosis when the amount of residual cancer cells is very small after treatment [10]. In the present patient, because it was possible that the balance was stable, the metastasis did not develop and was controlled in the appendix.

Most patients with metastases from cervical cancer are not curable by resection, but some have disease that is isolated to the lymph nodes or a limited area and are candidates for surgical resection. Complete resection of a metastatic appendiceal tumor without other metastases has no influence on the prognosis [4].

## Data Availability

The datasets supporting the conclusions of this article are included within the article and its additional files.

## Abbreviations

PET–CT: Positron emission tomography with computed tomography
CK7: Cytokeratin 7
CK20: Cytokeratin 20
ER: Estrogen receptor
PgR: Progesterone receptor
